# B-GATA transcription factors – insights into their structure, regulation, and role in plant development

**DOI:** 10.3389/fpls.2015.00090

**Published:** 2015-02-23

**Authors:** Carina Behringer, Claus Schwechheimer

**Affiliations:** Department of Plant Systems Biology, Technische Universität München Freising, Germany

**Keywords:** GATA, HAN-domain, LLM-domain, B-GATA, HANABA TARANU, GNC, GNL, CGA1

## Abstract

GATA transcription factors are evolutionarily conserved transcriptional regulators that recognize promoter elements with a G-A-T-A core sequence. In comparison to animal genomes, the GATA transcription factor family in plants is comparatively large with approximately 30 members. Here, we review the current knowledge on B-GATAs, one of four GATA factor subfamilies from *Arabidopsis thaliana*. We show that B-GATAs can be subdivided based on structural features and their biological function into family members with a C-terminal LLM- (leucine–leucine–methionine) domain or an N-terminal HAN- (HANABA TARANU) domain. The paralogous GNC (GATA, NITRATE-INDUCIBLE, CARBON-METABOLISM INVOLVED) and CGA1/GNL (CYTOKININ-INDUCED GATA1/GNC-LIKE) are introduced as LLM-domain containing B-GATAs from *Arabidopsis* that control germination, greening, senescence, and flowering time downstream from several growth regulatory signals. *Arabidopsis* HAN and its monocot-specific paralogs from rice (NECK LEAF1), maize (TASSEL SHEATH1), and barley (THIRD OUTER GLUME) are HAN-domain-containing B-GATAs with a predominant role in embryo development and floral development. We also review GATA23, a regulator of lateral root initiation from *Arabidopsis* that is closely related to GNC and GNL but has a degenerate LLM-domain that is seemingly specific for the *Brassicaceae* family. The *Brassicaceae*-specific GATA23 and the monocot-specific HAN-domain GATAs provide evidence that neofunctionalization of B-GATAs was used during plant evolution to expand the functional repertoire of these transcription factors.

## B-GATA TRANSCRIPTION FACTORS

GATA factors are evolutionarily conserved transcription regulators that were named after their DNA-binding preference to the consensus sequence W-GATA-R [W, thymidine (T) or an adenosine (A); R, guanidine (G) or adenosine (A); Reyes et al., 2004]. All GATA transcription factors from *Arabidopsis* have a type IV zinc finger with the consensus C-X_2_-C-X_17-20_-C-X_2_-C (C, cysteine; X, any residue) followed by a highly basic amino acid stretch (Reyes et al., 2004). While the zinc finger engages in hydrophobic interactions with the minor grove of the target DNA, the basic stretch interacts with the negatively charged phosphate backbone. Whereas all *Arabidopsis* GATAs have only one DNA-binding domain, several GATA transcription factors from rice, similarly to their animal counterparts, contain more than one zinc finger (Reyes et al., 2004).

The interest in GATA transcription factors from plants was originally instigated by the observation that GATA motifs are enriched in promoters of light-regulated genes and of genes controlled by the circadian clock (Arguello-Astorga and Herrera-Estrella, 1998). The interest in GATAs was further stimulated by the fact that the GATA factor AreA from the fungus *Aspergillus nidulans* is a key regulator of nitrogen signaling, which suggested that studies of plant GATAs may also lead to advances in understanding nitrogen signaling in plants (Daniel-Vedele and Caboche, 1993; Scazzocchio, 2000). In spite of this long-standing interest, only recently the identification and availability of mutants and overexpressors has allowed determining the function of these GATA factors in a biologically relevant context. Although in several cases functional redundancy between different GATA genes has rendered the identification of their biological functions difficult, it is now apparent that GATAs play a key role in a wide array of biological processes.

**FIGURE 1:**
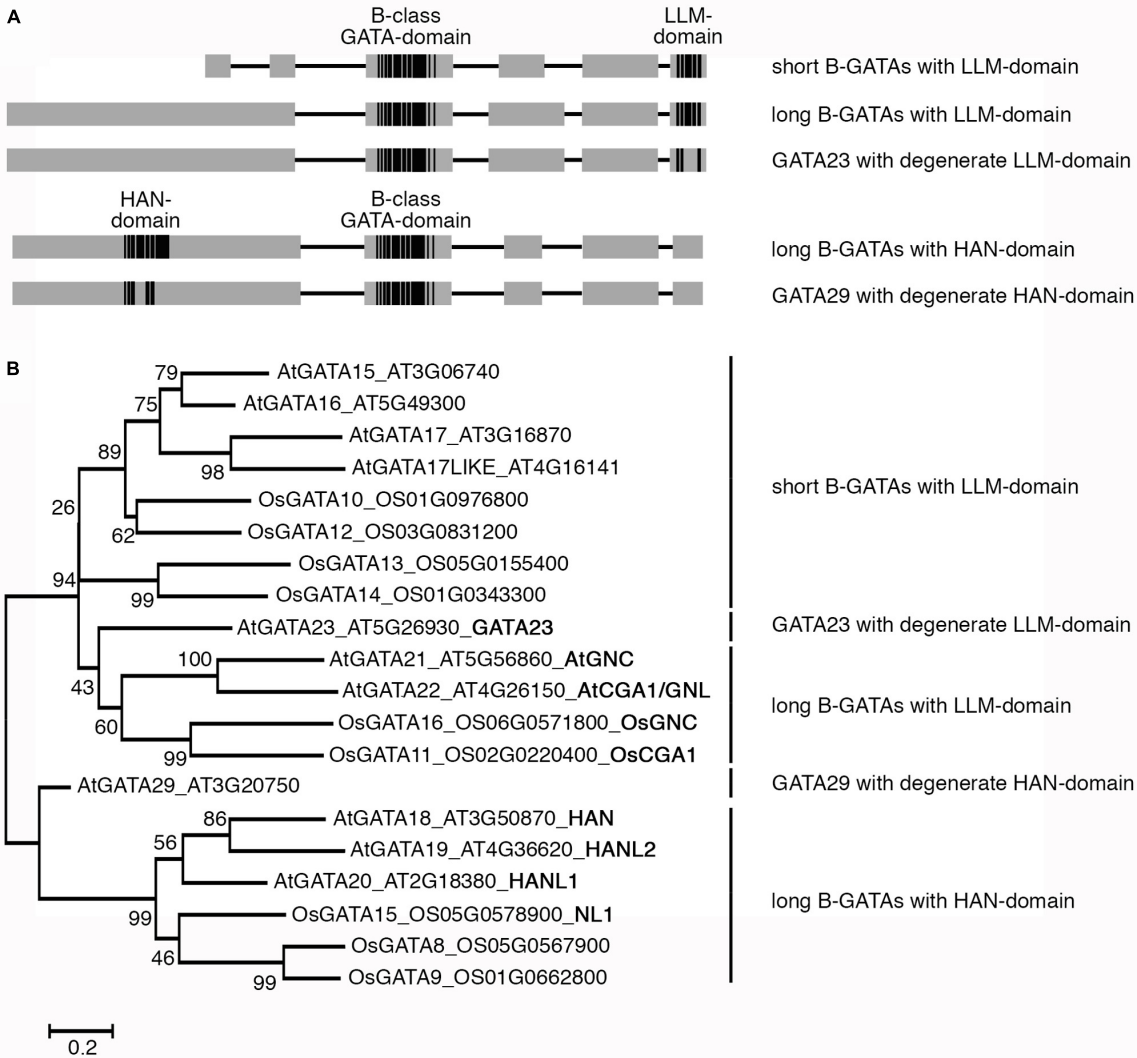
**Overview of B-class GATA transcription factors from *Arabidopsis thaliana* and rice (*Oryza sativa*). (A)** Schematic representation of B-class GATAs with their B-class GATA DNA-binding domain, the C-terminal LLM- (leucine–leucine–methionine) domain and the N-terminal HAN- (HANABA TARANU) domain. In *Arabidopsis*, B-GATAs with a degenerate HAN- or LLM-domain can be found as specified in subsequent Figures. Boxes represent protein regions with sequence similarity (gray) or high sequence conservation (black), lines represent protein regions with restricted sequence conservation. The schemes are not drawn to scale but reflect the presence of long and short proteins and the respective positions of the LLM- or HAN-domain. **(B)** Phylogenetic tree of B-GATAs from *A. thaliana* and rice (*O. sativa*). Where available, trivial names as introduced in the main text are provided (bold). The phylogenetic tree was generated using the Geneious R7 Software based on a MUSCLE alignment in MEGA6.06 using the following settings: Gap penalty, gap open -2.9, gap extend 0, hydrophobicity multiplier 1.2; interations, maximum iterations 8; clustering method, all iterations UPGMB and minimum diagonal length (lambda) 24. The Neighbor Joining tree was generated with the bootstrap method (1000 replications) using the Jones-Taylor-Thornton model using default settings. Bootstrap values are indicated by each node. Bar = 0.2 amino acid substitutions per site.

The knowledge about the identity of GATA factors from *Arabidopsis* and rice allowed subdividing the approximately 30 plant GATA factors into four conserved and distinct classes; class A through class D (Reyes et al., 2004). This classification was based on several criteria such as sequence conservation within the DNA-binding domain, the presence and absence of additional recognizable protein domains as well as the exon-intron structures of the respective genes. The focus of this review is on class B GATAs (B-GATAs), which can be subdivided into at least two functional subfamilies based on the presence of conserved domains. Whereas some B-GATAs contain a conserved LLM- (leucine–leucine–methionine) domain at their very C-terminus with an invariant L–L–M motif (Behringer et al., 2014) others contain a conserved HAN domain, which was first described in the *Arabidopsis* B-GATA HAN (HANABA TARANU; **Figure 1A**). LLM- and HAN-domain containing B-GATAs can be identified in all sequenced dicot and monocot species suggesting that they existed before the monocot-dicot divergence (**Figure 1B**; Behringer et al., 2014). Several members of the *Arabidopsis* B-GATA family have already been intensively studied: first, the paralogous GNC (GATA, NITRATE-INDUCIBLE, CARBON-METABOLISM INVOLVED) and CGA1/GNL (CYTOKININ-INDUCED GATA1/GNC-LIKE; hitherto GNL), representative B-GATAs with an LLM-domain; second, HAN and HANL (HAN-LIKE) proteins from *Arabidopsis* and monocots, B-GATAs with a HAN-domain. Furthermore, there are *Brassicaceae*-specific as well as monocot-specific B-GATAs that together provide evidence that the neofunctionalization of B-GATAs was used during plant evolution to expand their functional repertoire (**Figure 1**). In this review, we will summarize the current knowledge about B-GATAs, their structure, their regulation, and their role in plant development.

## *GNC* AND *GNL* – GROWTH REGULATORS DOWNSTREAM FROM MULTIPLE PHYTOHORMONE PATHWAYS

**FIGURE 2:**
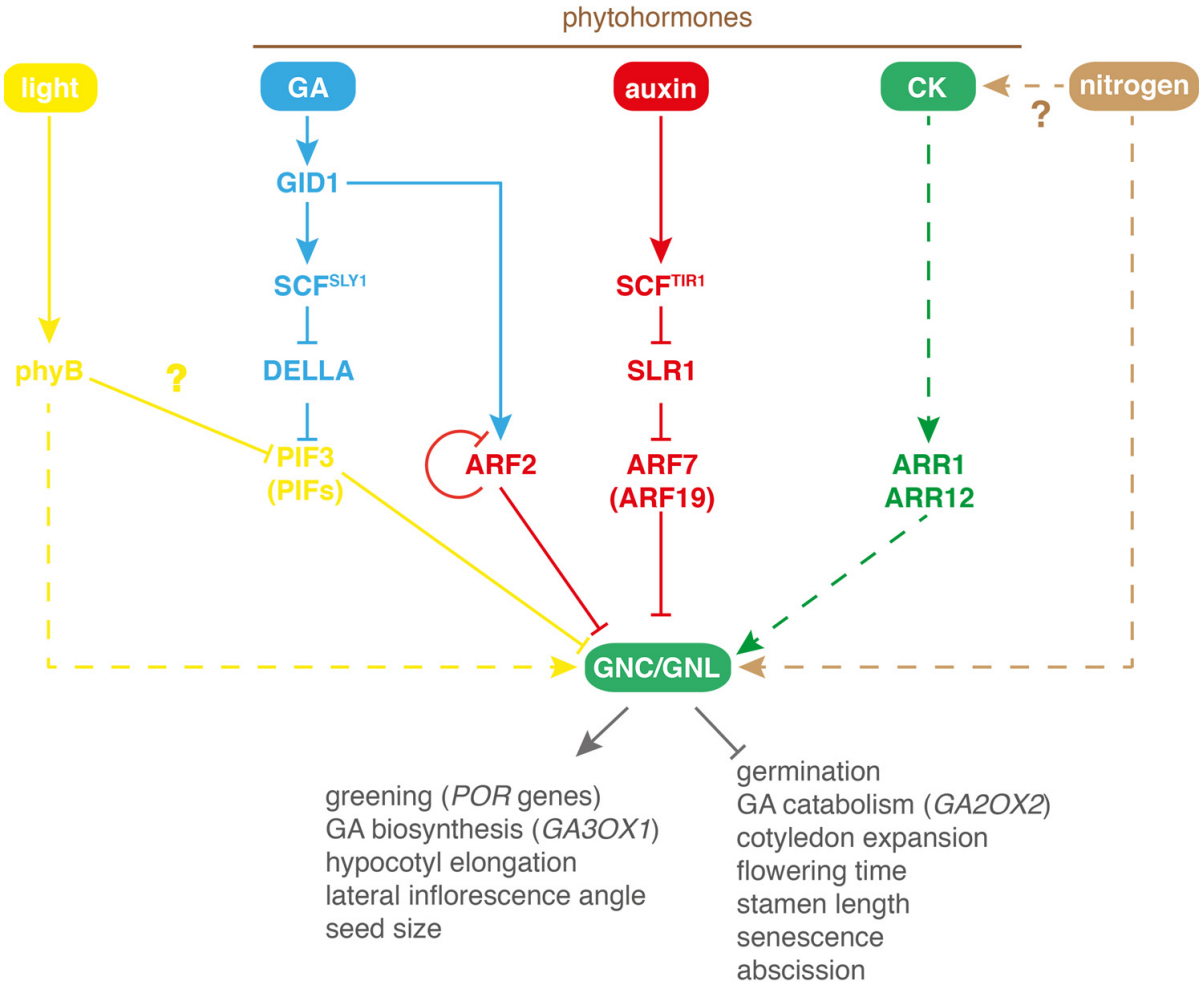
**Model of the different regulatory pathways regulating *GNC* and *GNL* transcription in *A. thaliana.*** phyB, phytochrome B; PIF, PHYTOCHROME INTERACTING FACTOR; GA, gibberellin; GID1, GIBBERELLIN INSENSITIVE DWARF1; SCF, SKP1-CULLIN-F-BOX protein type E3 ubiquitin ligase with the F-box protein SLY1 (SLEEPY1; SCF^SLY 1^) or TIR1 (TRANSPORT INHIBITOR RESISTANT1, SCF^TIR1^); ARF, AUXIN RESPONSE FACTOR; SLR1, SOLITARY ROOT1 (AUX/IAA protein); CK, cytokinin; ARR, *ARABIDOPSIS* RESPONSE REGULATOR. Continuous lines indicate direct and experimentally validated regulation; dashed lines indicate indirect regulation; question marks indicate proposed regulatory modes of action.

**FIGURE 3:**
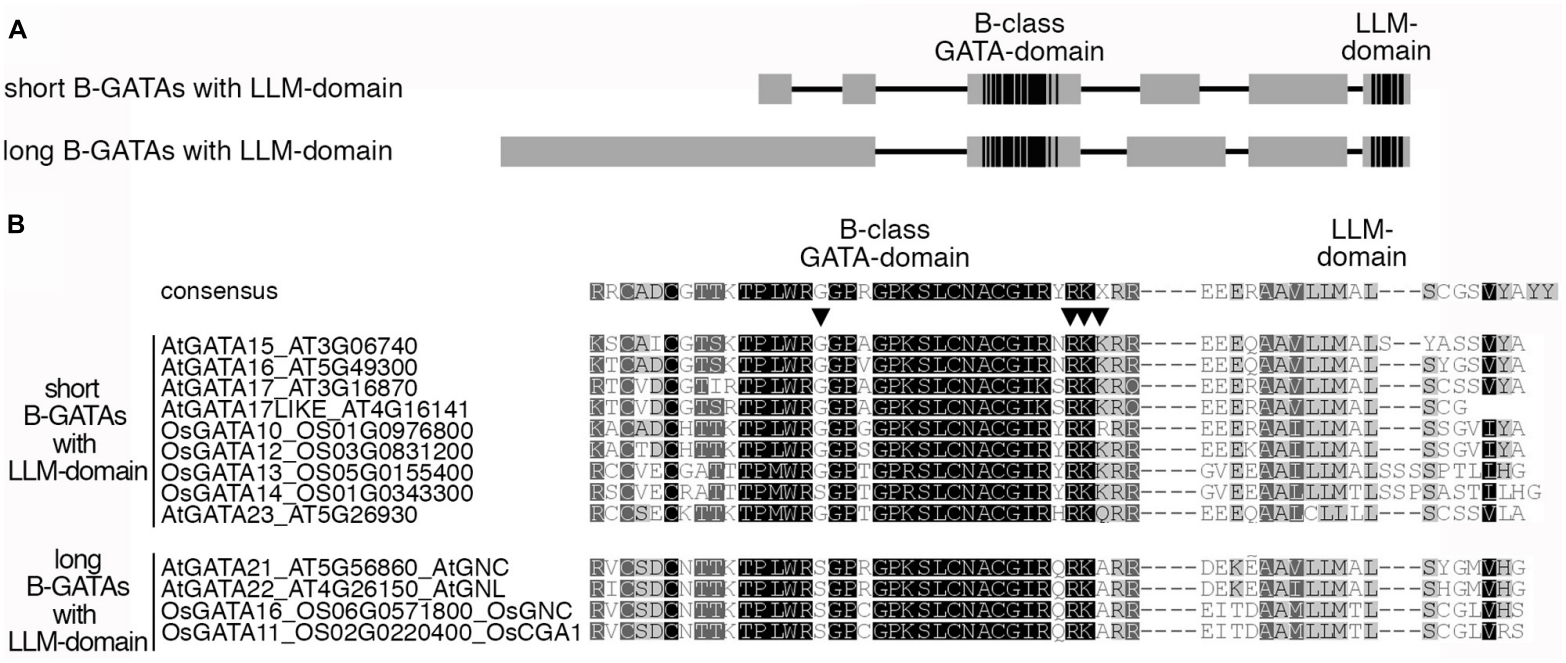
**LLM-domain containing B-GATAs from *Arabidopsis* and rice. (A)** Schematic representation of the short and long B-GATAs with the C-terminal LLM-domain. Boxes represent protein regions with sequence similarity (gray) or high sequence conservation (black), lines represent protein regions with restricted sequence conservation. **(B)** Sequence alignment of the GATA-domain and the LLM-domain of short and long B-GATAs with an LLM-domain from *Arabidopsis* and rice. The presence of the LLM-domain can already be predicted based on the sequence of the GATA domain. Conserved residues that allow distinguishing LLM-domain containing B-GATAs from other B-GATAs are marked with arrowheads.

**FIGURE 4:**
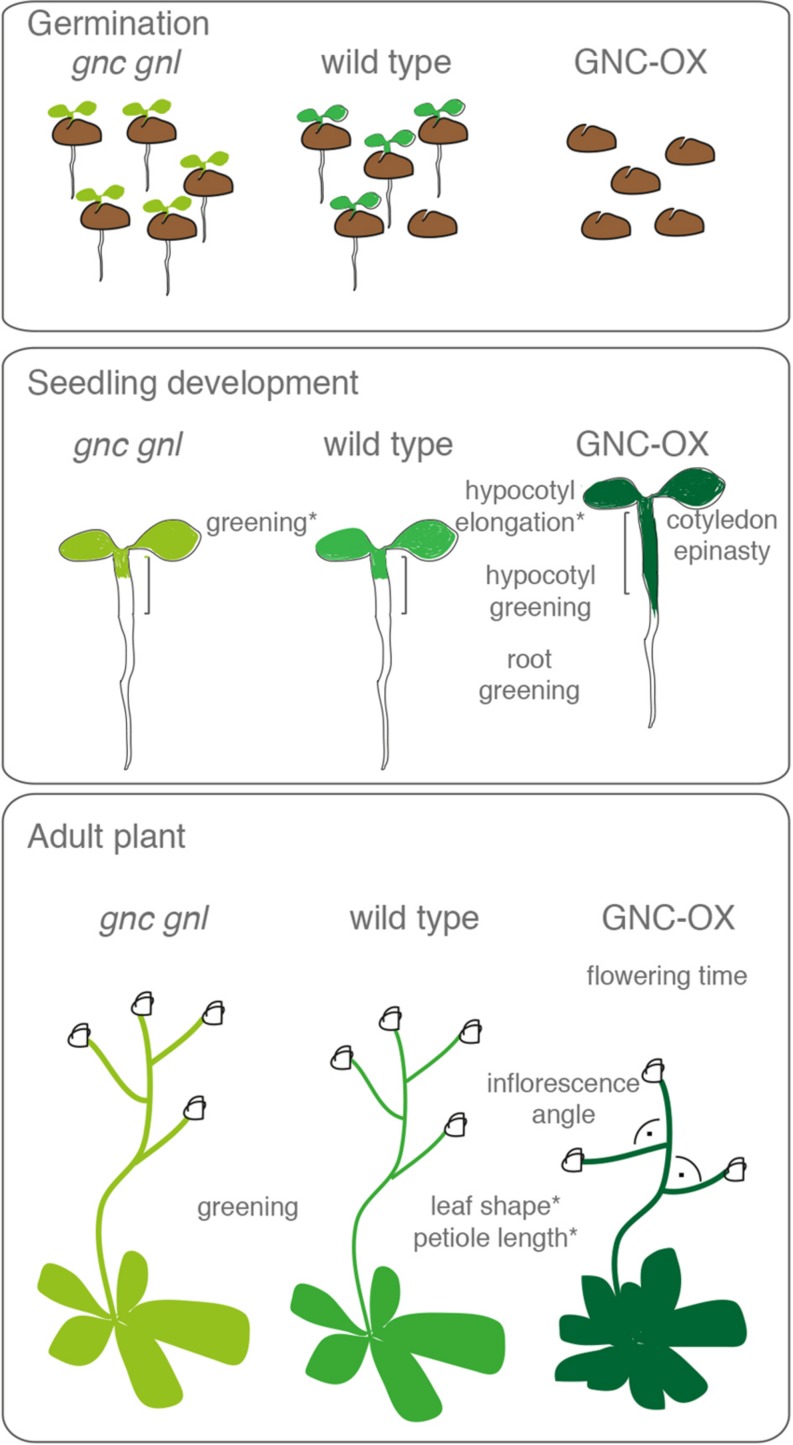
**Phenotypes of LLM-domain B-GATA loss-of-function mutants (*gnc gnl*) and overexpressors (GNC-OX) during germination, seedling development, and in adult plants.** Phenotypes that are marked with an asterisk are dependent on the presence of the LLM-domain and are not observed in overexpressors with a mutation or deletion of the LLM-domain (Behringer et al., 2014).

*GNC* and its paralog *GNL* (*GNC-LIKE*) had first been noted based on their transcriptionally regulation by nitrate (Wang et al., 2003; Price et al., 2004; Scheible et al., 2004; Bi et al., 2005; Kiba et al., 2005). *GNC* was subsequently identified as a gene required for proper chlorophyll accumulation and was designated *GATA, NITRATE-INDUCIBLE, CARBON-METABOLISM INVOLVED* based on the transcriptional regulation by nitrate and the misregulation of genes involved in carbon metabolism in the *gnc* mutant (Bi et al., 2005; **Figures 2 and 3**). GNL had initially been designated CGA1 based on its strong transcriptional regulation by cytokinin (CK) and light (Naito et al., 2007; **Figures 2 and 3**). Subsequent studies could then show that both GATAs, GNC, and GNL, contribute to the control of greening and also play a role in the regulation of plant development downstream of the hormones gibberellin (GA) and auxin (Richter et al., 2010, 2013b; **Figure 2**). Thus, these B-GATAs are under the control of multiple signaling pathways including nitrogen availability, several phytohormones as well as light (**Figure 2**). Common to at least some of these input pathways is that they modulate the greening of the plant, which is the most prominent phenotype not only in the loss-of-function mutant but also in the overexpressors of *GNC* and *GNL* (**Figure 4**). *GNC* and *GNL* were also identified as direct targets of the floral homeotic regulatory APETALA3 and PISTILLATA but the functional significance of this regulation remains to be explored (Mara and Irish, 2008).

*Arabidopsis* has six LLM-domain B-GATAs that can be subdivided into short and long family members. Comparative analyses suggest that the presence or absence of the LLM-domain correlates with functional differences between these B-GATAs but not protein length (**Figure 3**; Behringer et al., 2014).

### REGULATION OF *GNC* AND *GNL* TRANSCRIPTION BY GIBBERELLIN

Gibberellin signaling is mediated by interactions between GA and the GIBBERELLIN INSENSITIVE DWARF1 (GID1) GA receptors (Schwechheimer, 2014). GA-binding triggers the proteasomal degradation of DELLA proteins, negative regulators of GA signaling, via the E3 ubiquitin ligase SCF^SLY 1^ (SKP1 – CULLIN – F-BOX PROTEIN with the F-box protein SLEEPY1 [SLY1]) or related complexes (Dill et al., 2004). DELLAs interfere with the activities of other proteins, mainly transcription factors such as PHYTOCHROME INTERACTING FACTORS (PIFs). The GA-induced degradation of DELLAs relieves – in the case of the PIFs – their repressive interactions and allows PIFs to bind DNA (de Lucas et al., 2008; Feng et al., 2008). Studies on the role of *GNC* and *GNL* in GA signaling were instigated by the observation that their transcription is repressed by GA signaling. This transcriptional regulation of the two B-GATAs could be explained by the DELLA-dependent control of PIFs, notably PIF3, which directly binds to *GNC* and *GNL* promoter elements (**Figure 2**; Richter et al., 2010). Since PIF activity is not only negatively regulated by DELLA interactions but also by light, the previously reported light-induced transcription of *GNL* may be explained by the regulation of *GNL* by PIFs (Naito et al., 2007; **Figure 2**).

Mutants and overexpressors of *GNC* and *GNL* have a number of phenotypes that can be explained by defects in GA signaling in that they promote greening and hypocotyl elongation but repress germination and flowering (**Figure 4**). When compared to mutants with a strong GA pathway defect, the contribution of *GNC* and *GNL* to plant growth regulation is comparatively subtle. For example, the strong flowering time delay of the *ga1* mutant is only partially suppressed in *ga1 gnc gnl* (Richter et al., 2010, 2013a). In qualitative terms, this suppression is comparable to the suppression of the *ga1* phenotype by *DELLA* gene mutants from *Arabidopsis*. There, the loss of individual members of the five member *DELLA* gene family only partially suppresses *ga1* phenotypes, whereas the loss of multiple *DELLA* genes results in a strong genetic suppressions (Cheng et al., 2004; Cao et al., 2005). Taking into account that there are six presumably functionally redundant LLM-domain containing B-GATAs in *Arabidopsis* (**Figure 3**; Behringer et al., 2014), it could be envisioned that a stronger suppression of *ga1* can be achieved when all six LLM-domain B-GATAs are mutated in *ga1*.

### REGULATION OF *GNC* AND *GNL* BY AUXIN AND CROSS-TALK WITH GA SIGNALING

*GNC* and *GNL* are also transcriptionally repressed by AUXIN RESPONSE FACTOR2 (ARF2; Richter et al., 2013b). ARF2 belongs to the family of ARF transcription factors that have been subdivided into ARF+ that can bind the auxin-labile AUX/IAA repressors and ARF- that do not engage in such repressive interactions (Vernoux et al., 2011). AUX/IAA repressor abundance is negatively regulated by auxin through a specific SFC-type E3 ubiquitin ligase (Dharmasiri et al., 2005a,b). This regulation, however, only affects the activity of ARF+ and not that of ARF- such as ARF2.

*arf2* mutants share a number of phenotypes with *GNL* and *GNL* overexpression lines such as seed size, chlorophyll biosynthesis, stamen length, floral organ abscission, and senescence (Ellis et al., 2005; Okushima et al., 2005; Richter et al., 2013b; **Figure 4**). Interestingly, these *arf2* phenotypes are partially or fully suppressed in the presence of *gnc* and *gnl* loss-of-function mutants (Richter et al., 2013b). Thus, *arf2* mutant phenotypes may be explained by increased *GNC* or *GNL* transcript levels in *arf2* and the repressive activities of the GATAs in this mutant background. Indeed, *GNC* and *GNL* transcription is elevated in *arf2* mutants and ARF2 directly binds to the *GNC* and *GNL* promoters (Richter et al., 2013b).

Although ARF2 is an auxin regulation-independent ARF-, the transcriptional repression of *GNC* and *GNL* can be modulated by auxin. This suggested that also auxin-responsive ARF+ and AUX/IAAs may regulate *GNC* and *GNL* expression. Indeed, loss-of-function mutants of the ARF+ proteins ARF7 and its paralog ARF19 as well as gain-of-function mutants of their interacting AUX/IAA SLR1 (SOLITARY ROOT1) are phenotypically similar to *GNC* and *GNL* overexpressors. In line with a direct activity of ARF7 on the B-GATA promoters, an auxin-modulated binding of ARF7 to the *GNC* and *GNL* promoters could be demonstrated. Thus, *GNC* and *GNL* transcription is under the control of auxin- and AUX/IAA-independent (ARF2) as well as auxin- and AUX/IAA-dependent (ARF7) transcription factors (Richter et al., 2013b; **Figure 2**).

The observation that the two phytohormones, GA and auxin, repress the transcription of *GNC* and *GNL* suggested that modulation of the expression of the two GATAs would allow for a transcriptional cross-talk between these two pathways. In fact, several phenotypes of the *arf2* mutant could be suppressed by GA treatments or in the presence of a *spy* (*spindly*) mutation, which phenotypically mimics the phenotypes of plants with constitutively active GA signaling (Richter et al., 2013b). Thus, GA and auxin signaling converge on the transcriptional regulation of *GNC* and *GNL* and these two signals control at least in part the same growth responses (**Figure 2**).

The analysis of this GA-auxin cross-talk also resulted in the identification of two feedback regulatory mechanisms that contribute to the regulation of *GNC* and *GNL* expression (Richter et al., 2013b). First, ARF2 autoregulates its own transcription and thereby negatively feeds back on its own transcription as well as *GNC* and *GNL* regulation, and second, GA promotes ARF2 abundance by controlling ARF2 translation or by controlling the stability of a *de novo* synthesized and unknown GA-responsive protein involved in regulating ARF2 abundance (Richter et al., 2013b).

### *GNC* AND *GNL* PROMOTE GREENING DOWNSTREAM FROM CYTOKININ

*GNC* was isolated based on the greening defect of its loss-of-function mutant (Bi et al., 2005). Although such as greening defect is not visible (but quantifiable) in the *gnl* mutant, it is enhanced in the *gnc gnl* double mutant. *GNC* and *GNL* thus redundantly regulate greening, possibly together with other LLM-domain containing B-GATAs (**Figure 4**; Richter et al., 2010; Behringer et al., 2014). The greening phenotype of *GNC* and *GNL* overexpression lines correlates with the increased expression of the chloroplast localized *GLUTAMATE SYNTHASE*, *HEMA*, *GENOMES UNCOUPLED4*, and *PROTOCHLOROPHYLLIDE OXYDOREDUCTASE* genes as well as that of *PDV2 (PLASTID DIVISION2*; Richter et al., 2010; Hudson et al., 2011). At the same time, overexpression of *GNC*, *GNL,* or that of other B-GATAs induces a strong greening phenotype in tissues that normally do not contain significant numbers of chloroplasts such as the lower hypocotyl, the upper part of the root, and epidermal cells of cotyledons, and the hypocotyl (Richter et al., 2010; Chiang et al., 2012; Behringer et al., 2014). The role of the LLM-domain containing B-GATAs in the control of greening is conserved across species since the overexpression of LLM-domain containing B-GATAs from barley, tomato, or rice induces similar phenotypes when tested in *Arabidopsis* or rice, respectively (Hudson et al., 2011; Behringer et al., 2014). Taken together, LLM-domain containing B-GATAs are at least in some tissues sufficient to strongly promote greening.

**FIGURE 5:**
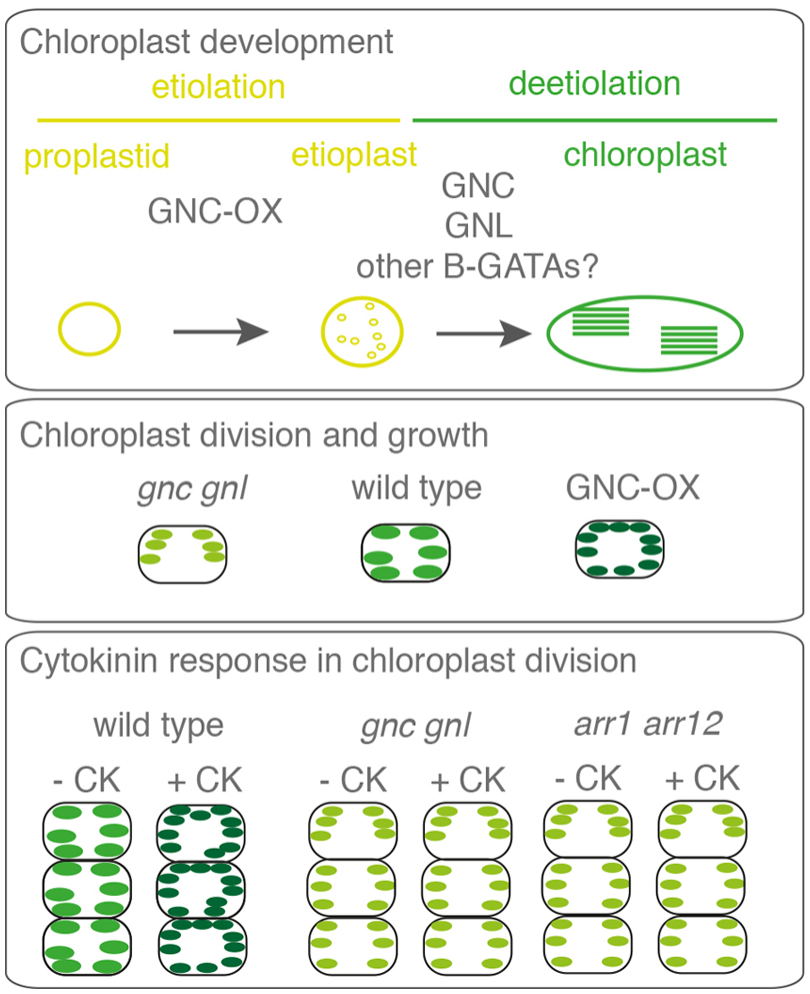
**Role of *GNC* and *GNL* as well as CK response in chloroplast division and growth.** *GNC* and *GNL* expression can promote the differentiation of proplastids to chloroplasts and the abundance of these B-GATAs also has an impact on chloroplast size. CK can promote changes in chloroplast division and chloroplast size that cannot be observed in the *gnc gnl* mutant or *arr1 arr12* mutant suggesting that *GNC* and *GNL* act downstream from the CK pathway (Chiang et al., 2012).

B-GATAs may control greening by promoting chlorophyll biosynthesis, chloroplast formation, or chloroplast size. In this regard, it is important to note that CK, which induces *GNC* and *GNL* expression, can promote greening in multiple developmental contexts (Kiba et al., 2005; **Figure 5**). CK induces chloroplast division by activating the expression of the chloroplast division regulators *PDV1* and *PDV2* in a manner that is dependent on the CK-induced regulator CRF2 (CYTOKININ RESPONSE FACTOR2; Okazaki et al., 2009). Although this increase in chloroplast division correlates with a reduction of chloroplast size, CK-treated plants have elevated chlorophyll levels (Okazaki et al., 2009). Furthermore, CK can promote greening ectopically in tissue that normally does not contain many chloroplasts including the upper part of the root (Kobayashi et al., 2012). Along these lines, the strong greening phenotype of the *GNC* overexpressors can be explained by an increased number of chloroplasts that is accompanied by the reduction of chloroplast size as it is typical for CK-treated seedlings (Chiang et al., 2012; **Figure 5**). Although the number of chloroplasts is not reduced, *gnc gnl* mutants have smaller chloroplasts in the hypocotyls and reduced chlorophyll levels in seedlings (Richter et al., 2010; Chiang et al., 2012). Additionally, ectopic expression of *GNC* promotes the differentiation of etioplasts from proplastids in dark-grown seedlings, which also can be correlated with an accelerated greening when etiolated seedlings are exposed to light (Chiang et al., 2012). CK treatment induces the expression of *GNL* but is less efficient in inducing the expression of *GNC* (Naito et al., 2007; Chiang et al., 2012). The type-B response regulators *ARABIDOPSIS* RESPONSE REGULATOR1 (ARR1) and ARR12 are important for this regulation since CK-induced gene expression of *GNL* is strongly compromised in *arr1 arr12* mutants where CK effects on chloroplast division are also compromised (Argyros et al., 2008; Chiang et al., 2012; **Figure 5**). Furthermore, *gnc gnl* mutants exhibit reduced CK sensitivity in chloroplast division (Chiang et al., 2012). Taken together these findings suggest that the greening defect of *gnc gnl* mutants is caused by their reduced CK-responsiveness and that this CK response requires the induction of *GNL* and possibly other B-GATAs through type-B ARRs. This adds LLM-domain containing B-GATAs to the list of transcription factors that can promote greening downstream from CK such as the previously mentioned CRF2 but also GLK2 (GOLDEN LIKE2; Fitter et al., 2002; Okazaki et al., 2009; Kobayashi et al., 2012). Although this has not been studied in detail, it may be that the effects of GA and auxin signaling on greening (Richter et al., 2010; Richter et al., 2013b) are, at least in part, also a consequence of the role of *GNC* and *GNL* on chloroplast division as demonstrated for CK signaling.

### CROSS-REPRESSIVE INTERACTIONS BETWEEN *GNC*, *GNL*, AND *SOC1* IN THE CONTROL OF FLOWERING TIME, GREENING, AND COLD TOLERANCE

**FIGURE 6:**
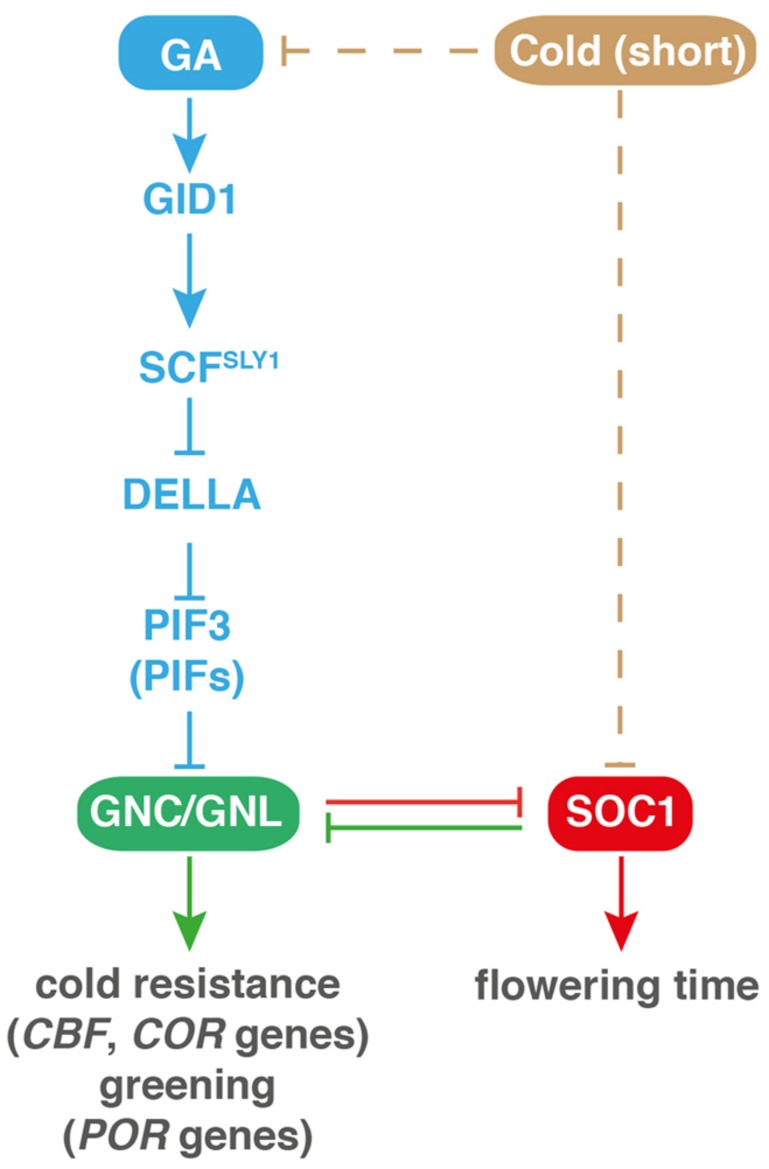
**Cross-repressive interactions between *GNC* and *GNL* with *SOC1* govern flowering time control, cold tolerance, and greening.** CBF, C-REPEAT/DRE BINDING FACTOR 1; COR, COLD RESPONSE; POR, PROTOCHLOROPHYLLIDE OXYDOREDUCTASE; SOC1, SUPPRESSOR OF THE OVEREXPRESSION OF CONSTANS1. See **Figure 2** legend for other abbreviations. Continuous lines indicate direct and experimentally validated regulation; dashed lines indicate indirect regulation.

*GNC* and *GNL* are flowering repressors. The contribution of these two GATAs to flowering time control can be observed in the GA-deficient late flowering mutant *ga1* where loss of *GNC* and *GNL* function promotes the flowering of *ga1* by about a month (Richter et al., 2013a). A dedicated analysis has placed these B-GATAs in the network around the flowering time regulator SOC1 (*SUPPRESSOR OF THE OVEREXPRESSION OF CONSTANS1*; Richter et al., 2013a). The MADS-box transcription factor SOC1 is a major regulator of flowering time in *Arabidopsis thaliana*. *SOC1* expression is under control of a number of flowering promoting inputs and *SOC1* expression is essential for floral induction in long day conditions (Samach et al., 2000; Yoo et al., 2005). In short-day conditions, *SOC1* is a major integrator of flowering time stimulation by GA (Blázquez and Weigel, 1999; Moon et al., 2003). Based on the central role proposed for *SOC1* in flowering time regulation, *SOC1* also qualified as a possible target of flowering time control downstream from *GNC* and *GNL*. Indeed, the promoter of *SOC1* is recognized by both GATAs and *SOC1* expression is strongly downregulated when the GATAs are overexpressed (**Figure 6**). In turn, when *SOC1* expression is uncoupled from *GNC* and *GNL* control in a *SOC1* overexpression line, the flowering repressive effects of *GNC* and *GNL* overexpression are suppressed. Thus, *GNC* and *GNL* act upstream of *SOC1* in flowering time control.

Curiously, the respective genetic interaction experiments also indicated that there may be an inverse relationship between *SOC1* and the GATAs in the control of other B-GATA-regulated responses that are not directly related to flowering time control (**Figure 6**). In fact, the genetic interaction experiments between *SOC1*, *GNC*, and *GNL* indicated that two other phenotypes of *soc1* mutants, enhanced greening and decreased cold tolerance, are suppressed in the absence of the *GNC* and *GNL* regulators (Richter et al., 2013a). Thus, cross-repressive interactions between these B-GATAs and SOC1 govern distinct biological processes.

## HAN-DOMAIN CONTAINING B-GATAs REGULATE EMBRYOGENESIS AND FLOWER DEVELOPMENT

**FIGURE 7:**
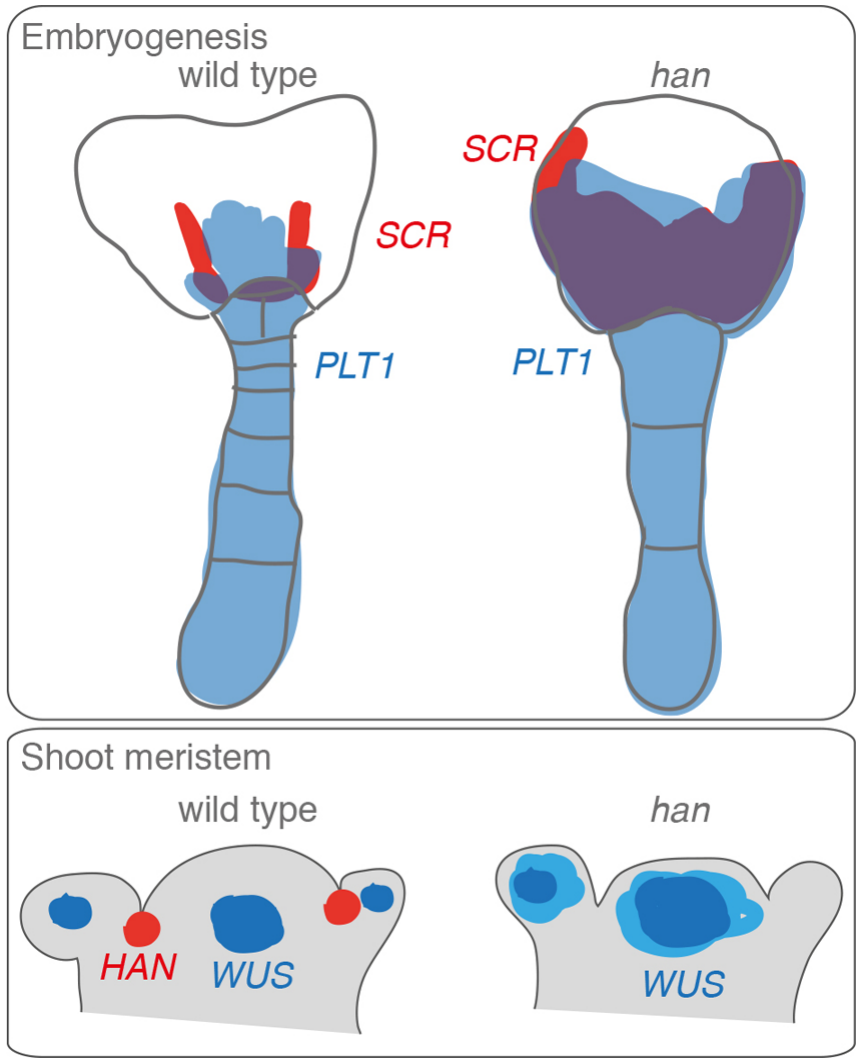
**Defects of *han* mutants during embryogenesis and shoot meristem formation.** *HAN* is expressed in the apical part of the embryo and defects in *HAN* expression lead to changes in the proembryo boundary formation that correlated with altered auxin distribution, altered *PIN* (*PIN-FORMED*) gene expression as well as altered expression of the marker genes *PLT1* (*PLETHORA1*) and *SCR* (*SCARECROW*; Nawy et al., 2010). *HAN* is expressed in the boundary between the different organs that originate from the shoot apical meristem and the loss of *HAN* results in a broadened expression of the *WUS* (*WUSCHEL*) marker.

**FIGURE 8:**
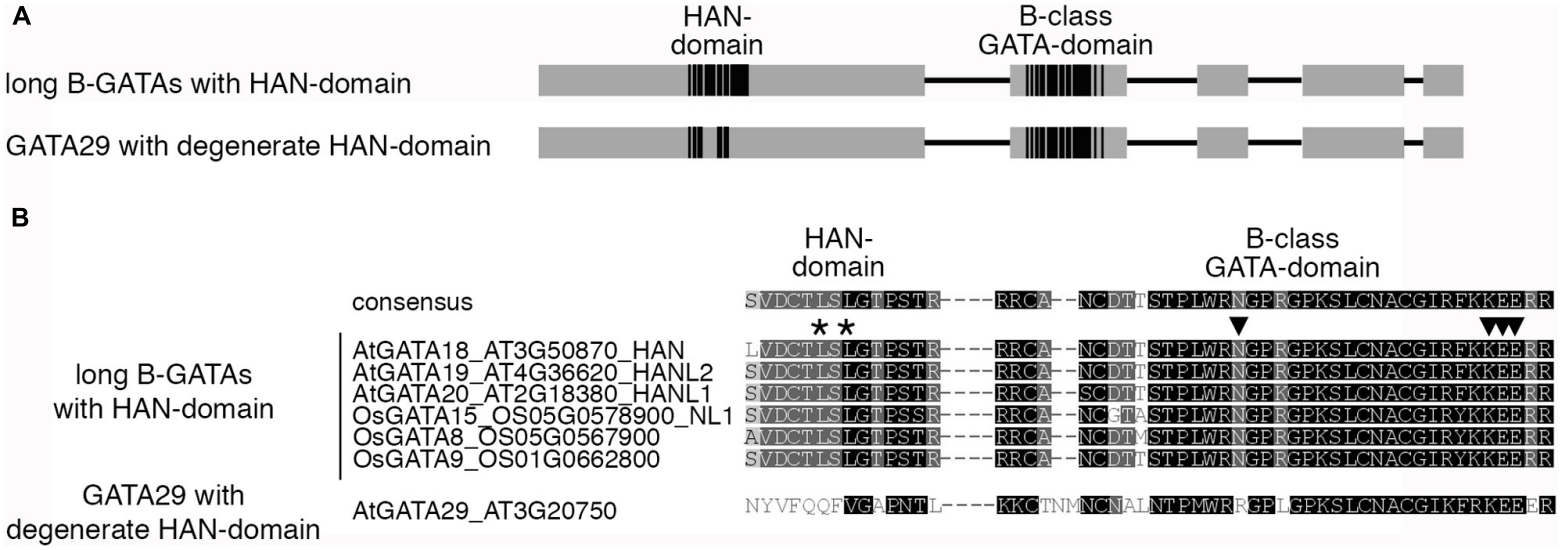
**HAN-domain containing B-GATAs from *Arabidopsis* and rice. (A)** Schematic representation of B-GATAs with an N-terminal HAN-domain. The HAN-domain of *Arabidopsis* GATA29 is degenerate and apparent orthologs of GATA29 cannot be retrieved in rice. Boxes represent protein regions with sequence similarity (gray) or high sequence conservation (black), lines represent protein regions with restricted sequence conservation. **(B)** Sequence alignment of the HAN-domain and the GATA-domain of HAN-domain containing B-GATAs from *Arabidopsis* and rice. The presence of the HAN-domain can already be predicted based on the sequence of the GATA domain. Conserved amino acids that allow distinguishing HAN-domain B-GATAs from other B-GATAs are marked with arrowheads. The asterisks mark residues in the HAN-domain as identified in loss-of-function mutant alleles of HAN family members providing evidence for the functional importance of this protein domain for protein function (Whipple et al., 2010; Kanei et al., 2012).

*HAN* (*HANABA TARANU*; Japanese for *floral leaf*; *TARANU*, Japanese for *not enough*) was independently identified in genetic screens as a mutant with altered floral organ identity (Zhao et al., 2004) and altered embryo patterning (Nawy et al., 2010; **Figure 7**). The HAN-domain, which is specific for this family of B-GATAs was first noted in HAN and its HAN-LIKE paralogs from *Arabidopsis* and later used to classify further B-GATAs as monocot-specific HAN-paralogs (**Figures 1 and 8**; Zhao et al., 2004; Whipple et al., 2010). The biological role of this B-GATA-specific domain is as yet unknown but may serve for interactions with other proteins. Whereas the overexpression of LLM-domain containing B-GATAs gives rise to a number of growth defects, most prominently the accumulation of chlorophyll at the base of the hypocotyl and hypocotyl elongation, *HAN* and *HANL2* overexpressors have different phenotypes, e.g., they accumulate less chlorophyll than the wild type and have normal hypocotyl length (Behringer et al., 2014). Thus, based on these criteria, HAN-domain B-GATAs are functionally distinct from LLM-domain containing B-GATAs.

### HAN – A REGULATOR OF EMBRYO DEVELOPMENT

During embryo development, *HAN* is required for the proper positioning of the proembryo boundary (**Figure 7**). *han* mutant embryos have several developmental defects, including a vacuolation of the lower tier cells of the embryo and a decrease in suspensor cell divisions (Nawy et al., 2010). The expression domains of embryonic markers for the suspensor and the lower tier are shifted apically in globular stage *han* embryos and this fits, in the case of the suspensor marker-positive cells, to their morphological resemblance to suspensor cells. The apical shift of auxin distribution in *han* mutant embryos and a broadening of the expression of the auxin response marker DR5:GFP (DR5:GREEN FLUORESCENT PROTEIN) are further indications for defects in proembryo boundary positioning in these mutants. Since lower tier cells normally give rise to the root, root formation is impaired in *han* embryos. *han* mutants fail to form an embryonic root and they are unable to undergo an essential cell division of the uppermost suspensor cell, the hypophysis that produces the quiescent center (QC). Interestingly, most *han* mutants can later recover from this defect and produce a root independently of the hypophysis at a later stage of embryo development (Nawy et al., 2010). It is thought that this rescue is the consequence of the coincidental expression of several prerequisites for QC formation: a local auxin maximum and the expression of the root regulators *PLETHORA*, *SHORT ROOT* and *SCARECROW* (Nawy et al., 2010). In addition to root development phenotypes, *han* mutants have also defects in cotyledon growth and initiation. *han* mutants sometimes have up to four cotyledons (Zhao et al., 2004). Ectopic root formation and altered *PLETHORA* expression were also observed in a *han* allele that strongly enhances the phenotype of mutants of the leaf development regulator *ANGUSTIFOLIA3* (Kanei et al., 2012).

Auxin is an actively transported hormone and its distribution within the embryo is mediated by auxin eﬄux carriers such as PIN1 (PIN-FORMED1) and PIN7 (Friml et al., 2003). In the wild type, auxin initially accumulates in the apical part and shifts to the suspensor preceding hypophyseal cell specification. This shift in auxin distribution correlates with a shift of PIN7 in the suspensor from the apical to the basal plasma membrane and a shift of PIN1 from being non-polarly distributed to being polarly distributed in the provascular cells of the proembryo (Friml et al., 2003). Both, the expression domain of *PIN1* as well as that of *PIN7* is shifted apically in *han* mutants and the ectopic expression of the *PINs*, at least that of *PIN1*, can find its explanation in a possibly direct transcriptional regulation of *PIN1* by HAN (Nawy et al., 2010). Thus, the *han* mutant phenotype may have its molecular cause in a misexpression of the PINs and consequently altered auxin distribution.

### HAN – A FLORAL MORPHOLOGY REGULATOR

HAN was first described based on the *han* mutants with altered shoot meristem morphology (Zhao et al., 2004; **Figure 7**). When compared to the wild type, *han* loss-of-function mutants have small flat shoot meristems, reduced numbers of floral organs in all four whorls as well as fused sepals. *HAN* is expressed between the meristem and between newly initiated floral organ primordia and in the boundaries between the different floral organ whorls. *HAN* overexpression, on the other side, results in delayed plant growth, disturbed cell divisions, and a loss of meristem activity. Taken together these findings suggest that HAN acts as a repressor of cell proliferation and that loss of this repressive function could lead to the reduced meristem size, which may be the cause for the reduced floral organ numbers and fused floral organs seen in its mutants (Zhao et al., 2004).

*HAN* expression surrounds the floral meristem cells and *HAN* interacts strongly with the *CLV* (*CLAVATA*) pathway (Zhao et al., 2004). In *Arabidopsis*, shoot meristem size is determined on the one side by the plasma membrane-resident receptor proteins CLV1 and CLV2 that are co-expressed in the outer layer of the shoot meristem as well as their putative peptide ligand CLV3 that is expressed in the underlying tissue layers. Defects in any of the three *CLV* genes results in enlarged shoot and floral meristems and the formation of an increased number of floral organs. The expression of CLV3 is negatively regulated by the homeobox-type transcription factor WUSCHEL (WUS) and *CLV3* expression overlies the expression domain of *WUS* suggesting, in combination with evidence from mutant analyses, that *WUS* controls meristem size by restricting *CLV3* expression as an essential ligand for the CLV1 and CLV2 receptor proteins. A *han* mutation combined with *clv* gene mutations resulted in increased inflorescence fasciation and increased floral abnormalities (Zhao et al., 2004). It has been proposed that the *HAN* gene is required to control WUS expression and reduced *WUS* expression as well as ectopic *WUS* expression may have a role in controlling floral meristem growth and repress floral organ primordium initiation (**Figure 7**). Alternatively, it may be envisioned that defects in nutrient or signal transport hinder meristem growth and floral organ development since *HAN* is also expressed early in provascular cells (Zhao et al., 2004). Interestingly HAN is also expressed in the boundaries between different whorls and between different floral organs suggesting that HAN could also act as a repressor of cell divisions. In this regard, there are some interesting parallels to the role proposed for HAN-related B-GATAs in bract suppression in monocots as will be discussed below.

Molecular analysis for transcription factor targets identifies *HAN* as a repression target of JAGGED (Schiessl et al., 2014). Genes acting downstream of HAN were also searched for using translational fusions between HAN and the glucocorticoid receptor, which allows for the glucocorticoid hormone-induced translocation of the HAN-GR fusion protein from the cytoplasm to the nucleus (Zhang et al., 2013). This analysis identified a range of floral development regulators as well as phytohormone-related genes as targets of HAN and suggested that HAN can act as a transcriptional activator and repressor. Interestingly, amongst the phytohormonal target genes are genes of those phytohormonal pathways that are known to regulate the expression of the LLM-domain B-GATAs *GNC* and *GNL* such as the *DELLA* genes of the GA pathway, *ARR* genes of the CK pathway, and *ARF* and *AUX/IAA* genes of the auxin pathway. Although the chosen experimental approach would have permitted to test for direct transcription targets by blocking *de novo* protein synthesis this possibility was not exploited. In conjunction with the fact that rather long time points (4 h up to 72 h) after glucocorticoid treatment were used for the sampling of the material and that many transcription factor genes were found to be regulated downstream from HAN in this experiment argues that the majority of these downstream genes could represent indirect rather than direct targets of HAN.

Among the genes that were found to be HAN-regulated according to this experiment were also HANL2, GNC as well as GNL. The transcriptional repression of these three genes suggested that their downregulation may be part of a negative feedback mechanism that serves to control B-GATA levels. Indeed, HAN was found to be able to bind to its own promoter as well as the promoter of *GNC*. Furthermore, genetic interaction studies using mutants of these B-GATAs found that mutant combinations of *han* with *hanl2*, *gnc,* and *gnl* mutations resulted in a strong decrease in the number of petals formed in these mutants, sepal fusion defects, fertility defects, as well as carpel abnormalities (Zhang et al., 2013). Also during embryogenesis, the combination of B-GATA mutations renders the previously described *han* embryogenesis defects more severe and embryos frequently terminate differentiation and form only clusters of cells (Zhang et al., 2013). Although the respective mutant analyses suffer from the weakness that mutations in the Columbia and Landsberg *erecta* backgrounds were combined and some of the observed defects may therefore be the result of these combinations, the genetic interplay between *HAN* and the other B-GATAs is also supported by the fact that HAN can homodimerize and interact with HANL2 as well as with GNC and GNL in yeast two-hybrid system (Zhang et al., 2013).

## MONOCOT-SPECIFIC HAN-PARALOGS

Whereas the formation of bract leaves is blocked in flowers of cultivated rice, maize, or barley, mutants from each of these species are known where the formation of such bract leaves is derepressed. In each case, the respective locus was identified and found to correspond to the *HAN* paralogs genes *NL1* (NECK LEAF1) from rice, *TSH1* (TASSEL SHEATH1) from maize and *TRD* (THIRD OUTER GLUME) from barley (Wang et al., 2009; Whipple et al., 2010; **Figure 1**). In line with the mutant phenotype, it could be shown that the expression of these *HAN*-domain B-GATAs is restricted to a cryptic bract in the zone where the suppression of bract formation is observed in the wild type. Interestingly, these B-GATAs form a monocot-specific subclade of HAN-domain B-GATAs indicating that these B-GATAs were recruited for the suppression of bract outgrowth specifically during monocot evolution. The apparent role as a repressor of bract growth also fits to the proposed function for HAN as a repressor of growth and cell cycle activities in the shoot meristem.

## GATA23 – A *Brassicaceae*-SPECIFIC B-GATA WITH A DEGENERATE LLM-DOMAIN

**FIGURE 9:**
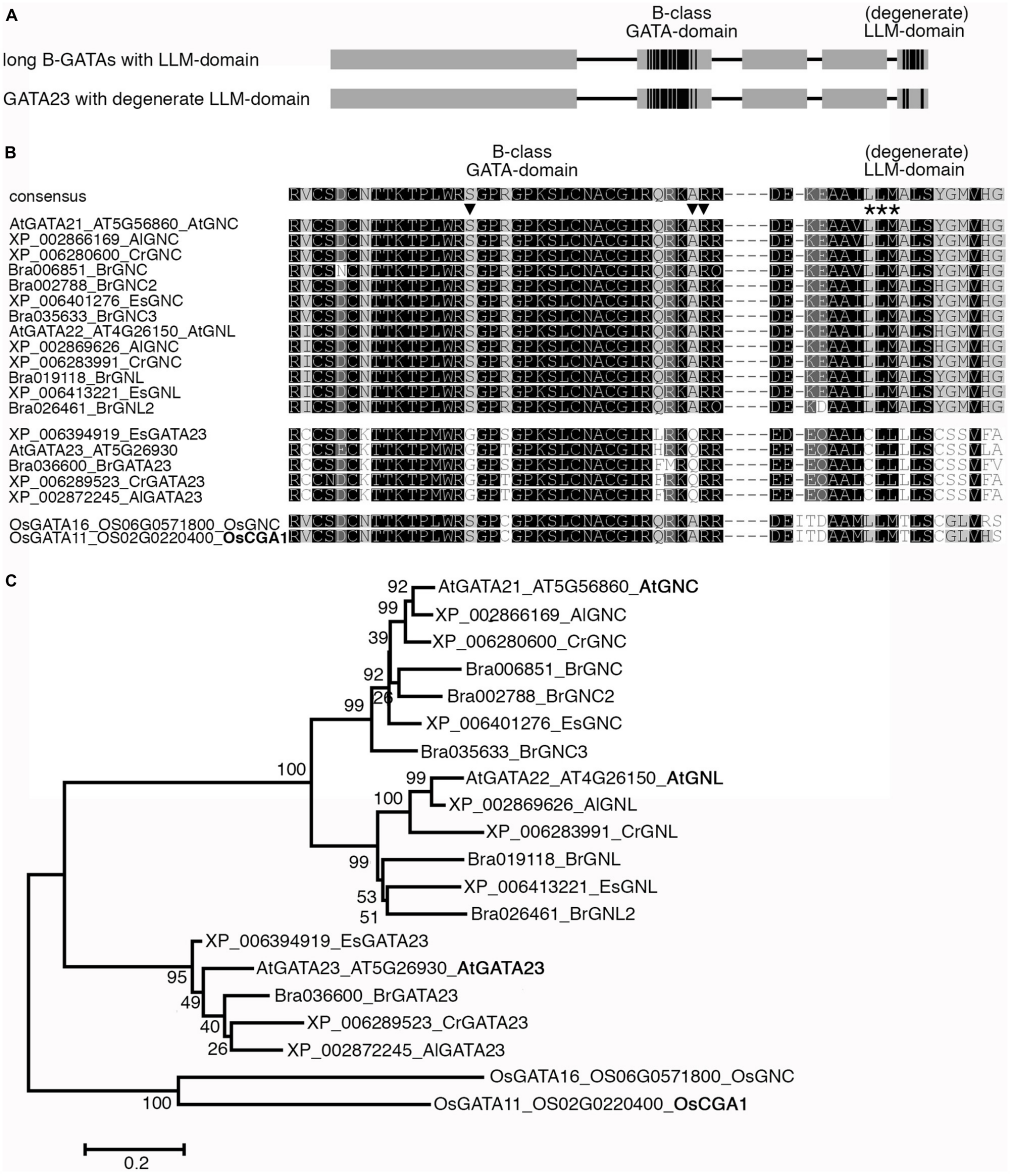
**GATA23 is specific for the *Brassicaceae*. (A)** Schematic representation of long B-GATAs with the C-terminal LLM-domain and GATA23 with a degenerate LLM-domain. Boxes represent protein regions with sequence similarity (gray) or high sequence conservation (black), lines represent protein regions with restricted sequence conservation. **(B)** Sequence alignment of the GATA-domain and the (degenerate) LLM-domain of the LLM-domain containing B-GATAs AtGNC and AtGNL as representatives for LLM-domain containing B-GATAs as well as AtGATA23 from *A. thaliana* (At) as a B-GATA with a degenerated LLM-domain and their predicted orthologues from other *Brassicaceae*: *A. lyrata* (Al), *Capsella rubella* (Cr), *Brassica rapa* (Br), *Eutrema salsugineum* (Es). Whereas the core LLM-motif is conserved among the GNC and GNL orthologues from the different *Brassicaceae* species and rice, it is divergent in the GATA23 B-GATAs. The triangles mark characteristic amino acid residues of the B-GATA domain that allow predicting the presence of the LLM-domain or a degenerate LLM-domain. Please note the conservation of these residues between the LLM-domain containing B-GATAs from the *Brassicaceae* and rice whereas the GATA23 orthologues are also divergent in these residues in the DNA-binding domain. **(C)** Phylogenetic tree of the B-GATAs shown in **(B)**. The phylogenetic tree was generated using the Geneious R7 Software based on a MUSCLE alignment in MEGA6.06 using the following settings: Gap penalty, gap open -2.9, gap extend 0, hydrophobicity multiplier 1.2; interations, maximum iterations 8; clustering method, all iterations UPGMB and minimum diagonal length (lambda) 24. The Neighbor Joining tree was generated with the bootstrap method (1000 replications) using the Jones-Taylor-Thornton model using default settings. Bootstrap values are indicated by each node. Bar = 0.2 amino acid substitutions per site.

Within both B-GATA subfamilies, there is one family member with a degenerate HAN- or LLM-domain, GATA29, and GATA23, respectively (**Figures 8 and 9**). Whereas there is no information about the role of GATA29, *GATA23* has been proposed to act in the root following its identification in a search for genes that are induced during the early steps of lateral root initiation (De Rybel et al., 2010). *GATA23* is specifically expressed in xylem pole pericycle cells before their first asymmetric division. Auxin accumulation is the first marker for lateral root founder cells and in line with an early role for *GATA23* during lateral root initiation, *GATA23* is auxin-induced. Since the expression of *GATA23* is impaired in gain-of-function mutants of the *AUX/IAA* gene *IAA28*, which is defective in lateral root formation, and since IAA28 interacts with several ARFs including the previously introduced ARF+ ARF7 and ARF19, a model was proposed, according to which auxin promotes lateral root initiation through degradation of the AUX/IAA IAA28 and subsequent ARF+-mediated *GATA23* expression. This model is supported by observations that lateral root initiation is partially suppressed in plants expressing an *GATA23* RNAi construct. The cell type specific expression of *GATA23*, in turn, correlates with increased lateral root initiations and uncoupling *GATA23* expression from auxin control also interferes with the normally regular spacing of lateral roots.

To what degree *GATA23* is important for lateral root initiation across species remains to be seen. Phylogenetic analyses have revealed that GATA23 from *Arabidopsis* belongs to a specific clade of B-GATAs with a degenerate LLM-domain that is closely related to GNC and GNL but functionally distinct (**Figures 1 and 9**; Behringer et al., 2014). At present, B-GATAs with the sequence features of GATA23 can only be identified in *Brassicaceae* and thus its function in non-*Brassicaceae* in lateral root initiation cannot be conserved outside of this family. Future research will have to elucidate the apparent functional diversification of these specific B-GATAs.

## OUTLOOK

Important advances have been made in understanding the role of B-GATA transcription factors in plant growth and development. Although there is now a comprehensive understanding of how the expression of these B-GATA genes is regulated at the transcriptional level, the knowledge about the identity of their target genes and cell type-specific activities is scarce. Candidate target genes of B-GATAs were genetically validated in a few exceptional cases only and, as yet, high quality transcription target analyses remain to be performed. Such experiments will be key to understand to what extent B-GATAs have overlapping and distinct transcription targets and should permit to delineate further to what extent differences in their expression domains or differences at the protein level contribute to their functional diversification.

Research on the LLM-domain containing B-GATAs has to date largely focused on the signaling events regulating their expression as well as on their role in the control of physiological processes such as greening and flowering. In turn, research on HAN-domain B-GATAs mainly focused on their role in the control of development. It can be anticipated that this apparent separation in the biological functions of B-GATAs between physiology and development will become more and more blurred in the future when developmental biologists will start studying LLM-domain B-GATAs and physiologists will study HAN-domain B-GATAs.

The fact that B-GATAs are unstable proteins that are turned-over by the 26S proteasome with a half-life of about 30 min is one interesting observation regarding all B-GATAs that requires further exploration (Behringer et al., 2014). It implies that there must be cognate E3 ubiquitin ligases that target these proteins for degradation. The identification of these E3 ligases will allow revealing cellular contexts where B-GATA abundance is differentially controlled and improve our understanding of B-GATA function.

The observation that different members of the plant B-GATA family, namely HAN, GNC, and GNL proteins, can interact with each other, could suggest that also other B-GATAs may act as homo- or heterodimers and may thus engage in interactions that could modulate their DNA-binding specificity or their function as transcriptional activators or repressors (Zhang et al., 2013; Behringer et al., 2014). Mammalian GATA factors interact with other transcription factors designated FRIEND OF GATA (FOG; Tsang et al., 1997; Fox et al., 1998), but obvious FOG homologues are not encoded by the plant genomes. Thus, this regulatory mechanism is most likely not conserved between animals and plants. It should be noted, however, that GATAs were found as interaction partners in yeast two-hybrid interaction analyses. First, *Arabidopsis* GNC was isolated as an interactor of the transcriptional co-regulator SIN3-LIKE1 (Bowen et al., 2010) and second, the LLM-domain B-GATA AtGATA16 appeared in a screen for proteins interacting with the co-repressor TOPLESS (Causier et al., 2012). Future research will have to reveal the biological significance of these interactions for GATA factor function.

## Conflict of Interest Statement

The authors declare that the research was conducted in the absence of any commercial or financial relationships that could be construed as a potential conflict of interest.
